# Impact of Soluble Schistosomal Egg Antigens on Type 1 Diabetes Mellitus in an Induced Diabetic Mouse Model

**DOI:** 10.1007/s11686-025-01035-w

**Published:** 2025-04-29

**Authors:** Mohammed Y. Shakra, Morsy R. M. Geneedy, Haitham Kh. Ahmad, Moamen A.I. Mazen, Mostafa E. Mostafa

**Affiliations:** 1 Department of Parasitology, Faculty of Medicine, Al-Azhar University, Damietta, Egypt; 2 Department of Parasitology, Faculty of Medicine, Al-Azhar University, Cairo, Egypt; 3 Department of Parasitology, Faculty of Medicine, Al-Azhar University, Assuit, Egypt

**Keywords:** *Schistosoma mansoni*, Streptozotocin, T1D, SEA, Cytokines

## Abstract

**Purpose:**

This study aimed to investigate the effects of *Schistosoma mansoni* soluble egg antigen (SEA) on type 1 diabetes (T1D) in a streptozotocin (STZ)-induced diabetic mouse model.

**Methods:**

The study examined the effects of Schistosoma mansoni soluble egg antigen (SEA) on type 1 diabetes (T1D) using a mouse model, involving 50 mice divided into three groups: a healthy control group receiving phosphate-buffered saline (PBS), a diabetic control group with STZ-induced T1D also receiving PBS, and a diabetic treated group receiving SEA. Biochemical and immunological analyses were conducted on blood samples collected at four and eight weeks post-treatment to assess metabolic markers like blood glucose and insulin levels, as well as immune markers including TNF-α, TGF-β, FOXp3, IL-4, and IL-10.

**Results:**

SEA treatment induced early immune modulation at four weeks and sustained metabolic and immunological improvements at eight weeks, marked by increased regulatory T cells (elevated FOXp3), activation of immunosuppressive pathways (increased TGF-β), reduced inflammation (decreased TNF-α), a shift to an anti-inflammatory Th2 response (elevated IL-4 and IL-10), improved glycemic control, lower blood glucose levels, and higher insulin levels.

**Conclusion:**

SEA exhibits potential therapeutic effects against T1D by modulating immune responses, promoting Th2 polarization, and increasing regulatory T cell activity. This immunological shift reduces systemic inflammation and enhances glycemic control.

## Introduction

In schistosomiasis, several biomolecules are released from the adult, larva and egg stages of the *Schistosoma* parasite into their host. These molecules can induce activation and modulation of innate and adaptive immune responses, and they also allow the parasite to evade host defence mechanisms. In both human and animal experimental models soluble egg antigens stimulate Th2 response during *Schistosoma* egg laying [1].

As the *Schistosome* infection progresses, the host immune response becomes polarized. It begins with a T helper type 1 (Th1) response against adult and larval stages of parasites characterized by the production of pro-inflammatory cytokines such as tumour necrosis factor-alpha (TNF-α), interferon-gamma (IFN-γ), and interleukin-12 (IL-12). A switch to a powerful Th2 response is induced by egg antigens, leading to increased levels of interleukins IL-4, IL-5, IL-9, and IL-13. This Th2 response is regulated by regulatory T cells (Tregs). In the chronic phase, interleukin-10 (IL-10) and transforming growth factor-beta (TGF-β) downregulate the inflammation but also lead to tissue damage, such as fibrosis and granuloma formation [2].

The Th1 immune response is also involved in the pathophysiology of autoimmune disorders. The immunomodulatory effects of *Schistosoma* infection produce the cytokine IL-10, which can inhibit the production of pro-inflammatory mediators like IFN-γ, TNF-α, and nitric oxide helping in controlling autoimmune diseases [3].

Type 1 diabetes mellitus (T1D) is a metabolic condition marked by decreased or absent insulin production, which raises blood glucose levels. About 537 million people worldwide currently are diabetic; by 2030, that figure might increase to 643 million, or 11.3% of the world’s population. By 2045, it is expected to reach 930 million, this raises the risk of both microvascular and macrovascular disorders [4].

Type 1 diabetes (T1D) is an organ-specific autoimmune disease that is brought on by the immune system targeting and destroying the pancreatic beta cells that produce insulin. Since T1D is a Th1-mediated autoimmune illness, diabetes could be avoided if the immune system were to be reoriented or adjusted toward Th2. Tregs proliferation and Th1 to Th2 shifting are two immunomodulatory pathways in helminthic diseases that help in protection against T1D [5].

Reduced exposure to helminths may be linked to the increasing incidence of Type 1 Diabetes (T1D), as suggested by the hygiene hypothesis. Originally proposed by Strachan in 1989 to explain the relationship between cleanliness and allergies, this hypothesis has evolved to include the role of helminth-mediated immunoregulation in protecting against autoimmune diseases like T1D in both human and animal research [6]. Since there is currently no cure for type 1 diabetes, daily or continuous subcutaneous insulin injections are used to regulate blood sugar levels. The search for novel therapeutics is critical. According to new research, parasitic worms, or helminths, may be able to reduce glycaemia and alleviate underlying inflammation [7].

As with a live *Schistosome* infection, SEA can interact with dendritic cells (DC), macrophages, natural killer T (NKT) cells, eosinophils, and basophils to activate and modulate the innate immune response. It can also act on T cells to activate the adaptive immune system, which leads to the downregulation of pro-inflammatory cytokines and the upregulation of anti-inflammatory cytokines [8].

Administration of *Schistosoma japonicum* soluble egg antigen (SEA) reduced blood glucose levels and the incidence of diabetes in non-obese diabetic (NOD) mice, suggesting a protective effect against type 1 diabetes. SEA increases the frequency of splenic regulatory T cells (Tregs) and the secretion of interleukins IL-4 and IL-5, promoting a T helper 2 (Th2) immune response, which contrasts with the Th1 response typically involved in autoimmune diabetes. SEA can maintain immune tolerance, prevent autoimmune responses, and prevent type 1 diabetes [9].

Based on previous studies, we investigate the effects of soluble *Schistosomal* egg antigens on biochemical and immunological parameters in type 1 diabetes mellitus using an induced diabetic mouse model.

### Materials and Methods

An experimental case-control study was conducted on fifty male C57BL/6J pathogen-free mice, weighing 18–25 g and aged 4 weeks. It was purchased from Theodor Bilharz Research Institute in Giza, Egypt. The experiments were conducted at the Al-Azhar Department of Parasitology, during the period from March to October 2024. They were housed in cages in conditioned rooms at 27 °C and fed on a balanced dry food with 14% protein, and sterile water. A 12-hour light/12-hour dark cycle was used to house the mice. All procedures and animal handling methods were approved by the Helsinki Declarations and the Ethics Committee of the Faculty of Medicine at Al-Azhar University in Cairo, Egypt.

### Animal Model

Cercarial shedding was carried out on snails (*Biomphalaria alexandrina*) infected with *Schistosoma mansoni* gained from Theodore-Bilharz Research Institute in Giza, Egypt. A subcutaneous injection of approximately 1000 cercariae per rabbit was used to infect two New Zealand rabbits [10]. For confirmation of infection, parasitological and histopathological examinations were done for the detection of eggs. Fecal pellets were collected from infected rabbits at 35th days post-infection and subjected to parasitological examination using direct wet mount and formol-ether concentration method [11]. Liver specimens were sectioned at a thickness of 5 μm, embedded in paraffin blocks, fixed in 10% formalin, and stained with hematoxylin and eosin [12].

### *Schistosomal Mansoni* Soluble Egg Antigen Preparation

*Schistosome* eggs were extracted from rabbit livers six weeks after infection. Following the euthanasia of the infected rabbits, the livers were removed, mashed, and rinsed with a 1.2% sodium chloride solution to prevent egg hatching. For five hours, the liver homogenate was trypsinized on an incubator shaker set at 200 rpm and 37 °C. The homogenate was then filtered through nylon nets and rinsed with a 1.2% sodium chloride solution. After centrifuging the homogenate, the precipitate was aseptically frozen at -20 °C. *Schistosoma mansoni* eggs were lyophilized into powder after being frozen for a week and then sterilized by ultraviolet light [13].

### Induction of Type 1 Diabetes Mellitus (T1D)

Streptozotocin (STZ), purchased from Sigma Aldrich^®^ (USA), was dissolved in freshly prepared 0.1 M citrate buffer at pH 4.5. After a six-hour fasting period, STZ was administered at 150 mg/kg intraperitoneally to induce Type 1 Diabetes Mellitus [14]. Three days later, blood glucose levels were assessed with a glucometer. Mice that had serum blood glucose levels > 200 mg/dL were identified as diabetic and chosen for further study.

### Experimental Design

Group 1: HC (Healthy Control): 10 healthy mice were injected intraperitoneal with 0.1 ml of sterile phosphate-buffered saline (PBS) twice weekly for 4 weeks.

Group 2:DC (Diabetic control): included 20 mice STZ induced T1D and were injected intraperitoneally with 0.1 ml of sterile phosphate-buffered saline (PBS) twice weekly for 4 weeks.

Group 3:DT (Diabetic treated): included 20 mice STZ induced T1D and were injected intraperitoneally with 50 µg/mouse of SEA twice weekly for 4 weeks [9].

### Samples Collection

Four weeks after treatment blood samples were taken by puncturing the retroorbital sinus. Four weeks later(eight weeks after treatment) another blood samples were taken. The blood samples were centrifuged for 10 min at 2000 rpm to obtain serum. The serum samples were kept in a freezer at -20 °C until different parameters were assessed. Every mouse group underwent biochemical and immunological testing.

### Biochemical Parameters

The blood glucose levels were assayed in all groups by standard automated enzymatic techniques by using a colourimetric kit purchased from Biodiagnostic, Dokki, Giza, Egypt. The insulin levels were assayed in all groups by using ELISA kits obtained from MyBioSource Co., San Diego, California, USA (Cat. #: MBS038565).

### Immunological Parameters

The assay of cytokines TNF-α, IL-4, IL-10, TGF-β, and FOXp3 in serum samples was conducted using commercial ELISA kits from MyBiosource Co. (San Diego, California, USA) according to the manufacturer’s instructions. The specific kits utilized were TNF-α (Tumor Necrosis Factor Alpha) (Cat. #: MBS8820376), IL-4 (Interleukin 4) (Cat. #: MBS2504956), IL-10 (Interleukin 10) (Cat. #: MBS175883), TGF-β (Transforming Growth Factor Beta) (Cat. #: MBS160136), and FOXp3 (Forkhead Box P3) (Cat. #: MBS2510062). The optical density (OD) of the plates was measured at 450 nm using an ELISA reader (Bio-Rad mod. 680).

### Statistical Analysis

Software IBM-SPSS 18 (IBM-SPSS Inc., Chicago, IL, USA) was used to analyze coded and computerized data. Means, medians, ranges, frequencies, percentages, and standard deviations are examples of descriptive data. Continuous variables were checked for normality using the Shapiro-Wilk test, and variables with more than two categories were examined for mean differences using the ANOVA test. A P value < 0.05 was considered significant.

### Results

In this experimental case-control study, we investigated the effects of *Schistosoma mansoni* soluble egg antigen (SEA) on type 1 diabetes (T1D) using an induced diabetic mice model.

Table 1 shows the effect of SEA injection on the level of FOXp3 among the studied groups over the study period. Non-significant difference was observed (*p* = 0.182) at 4 weeks post-treatment. On the contrary, at 8 weeks post-treatment, there was a significant difference (*p* < 0.001) in the mean FOXp3 level between groups i.e., Diabetic treated group had a higher FOXp3 mean level (6.4 ± 1.8 ng/ml) compared to healthy control (1.9 ± 0.3 ng/ml, *p* < 0.001) and diabetic control (2.2 ± 1.2 ng/ml, *p* < 0.001). Unlikely, an insignificant difference was found between both control groups (*p* = 0.842). For within-group comparisons, the increase in FOXp3 levels was significant only in the diabetic-treated group (*p* < 0.001).

**Table 1:** Effect of SEA treatment on the FOXp3 biomarker among studied groups

	Healthy Control (1) (*n* = 10)	Diabetic Control (2) (*n* = 20)		Diabetic Treated (3) (*n* = 20)	*P*-value*
FOXp3 (ng/mL)					
• 4-weeks Post-	2.1 ± 0.6	1.8 ± 0.9		2.8 ± 2.5	= 0.182
P-value**	1 vs. 2 = 0.893	2 vs. 3 = 0.165		1 vs. 3 = 0.565	
• 8-weeks Post-	1.9 ± 0.3	2.2 ± 1.2		6.4 ± 1.8	< 0.001
P-value**	1 vs. 2 = 0.842	2 vs. 3 < 0.001		1 vs. 3 < 0.001	
P-value***	= 0.774	= 0.699		< 0.001	

A two-way ANOVA test was used to compare the mean difference *between groups **Pairwise ***within the group

As shown in Table 2, there was a significant difference in the mean TGF-β level between groups (*p* < 0.001) at 4 weeks post-treatment i.e., Diabetic treated group had a higher TGF-β mean level (34.5 ± 4.8 ng/ml) compared to healthy control (10.6 ± 2.3 ng/ml, *p* < 0.001) and diabetic control (22.4 ± 5.7 ng/ml, *p* < 0.001). Likewise, there was a significant difference between both controls (*p* < 0.001). Moreover, at 8 weeks post-treatment, there was a significant difference (*p* < 0.001) in the mean TGF-β level between groups i.e., Diabetic treated group had a higher TGF-β mean level (96.4 ± 6.7 ng/ml) compared to healthy control (25.6 ± 5.2 ng/ml, *p* < 0.001) and diabetic control (13.1 ± 2.9 ng/ml, *p* < 0.001). Likewise, there was a significant difference between both controls (*p* < 0.001). For within-group comparisons, the increase in TGF-β level was only significant for the diabetic-treated group (*p* < 0.001).

**Table 2:** Effect of SEA treatment on TGF-β biomarker among studied groups

	Healthy Control (1) (*n* = 10)	Diabetic Control (2) (*n* = 20)		Diabetic Treated (3) (*n* = 20)	*P*-value*		
TGF-β (pg/mL)							
• 4-weeks Post-	10.6 ± 2.3	22.4 ± 5.7		34.5 ± 4.8	< 0.001		
P-value**	1 vs. 2 < 0.001	2 vs. 3 < 0.001		1 vs. 3 < 0.001			
• 8-weeks Post-	13.1 ± 2.9	25.6 ± 5.2		96.4 ± 6.7	< 0.001		
P-value**	1 vs. 2 < 0.001	2 vs. 3 < 0.001		1 vs. 3 < 0.001			
P-value***	= 0.824	= 0.789		< 0.001			

A two-way ANOVA test was used to compare the mean difference *between groups **Pairwise ***within the group

**Fig. 1:**
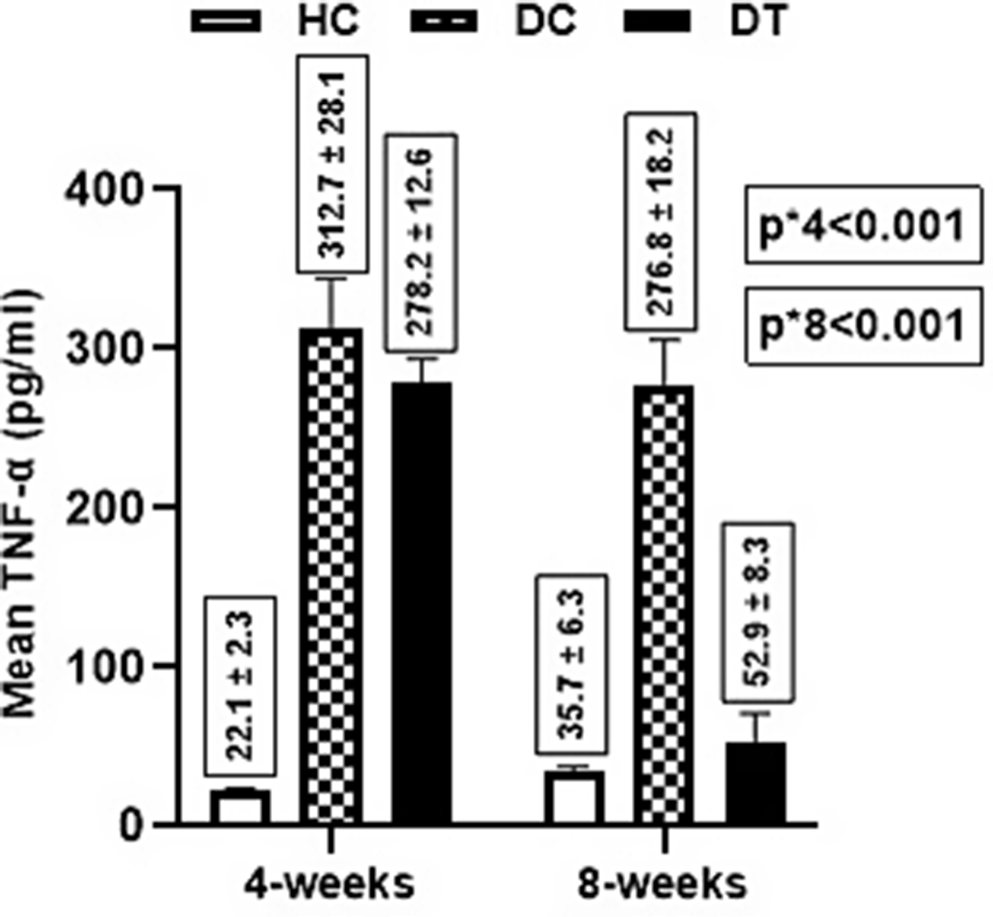
**Effect of SEA on the TNF-α Level between Groups.** P-value** 1 vs. 2 < 0.001 2 vs. 3 < 0.001 1 vs. 3 = 0.691 P-value*** = 0.598 = 0.446 < 0.001. A two-way ANOVA test was used to compare the mean difference *between groups **Pairwise ***within the group

For the TNF-α level, there was a significant difference in the mean TNF-α level between groups (*p* < 0.001) at 4 weeks post-treatment i.e., the healthy control(HC) group had lower TNF-α mean level (22.1 ± 3.2 pg/ml) compared to both diabetic control(DC) (312.7 ± 28.1 pg/ml, *p* < 0.001) and diabetic treated(DT) (278.2 ± 12.6 pg/ml, *p* < 0.001). Contrarily, an insignificant difference was found between both diabetic groups. (*p* = 0.298). Further, at 8 weeks post-treatment, there was a significant difference (*p* < 0.001) in the mean TNF-α level between groups i.e., the Diabetic control group had a higher TNF-α mean level (276.8 ± 18.2 pg/ml) compared to healthy control (35.7 ± 6.3 pg/ml, *p* < 0.001) and diabetic treated (52.9 ± 8.3 pg/ml, *p* < 0.001). In contrast, there was a non-significant difference between healthy control and diabetic-treated groups (*p* = 0.691). For within-group comparisons, the reduction in TNF-α level was only significant for the diabetic-treated group (*p* < 0.001) (Fig. 1).

**Fig. 2:**
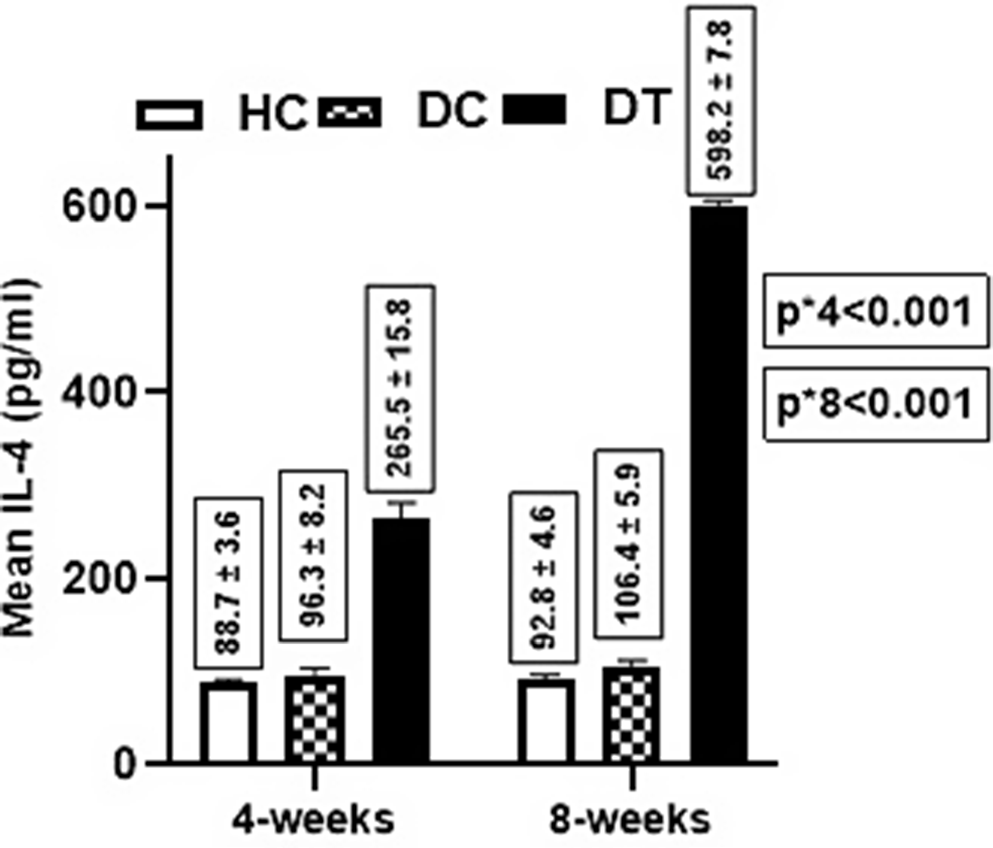
Effect of SEA on the IL-4 Level between Groups. P-value** 1 vs. 2 = 0.159 2 vs. 3 < 0.001 1 vs. 3 < 0.001 P-value*** = 0.667 = 0.508 < 0.001. A two-way ANOVA test was used to compare the mean difference *between groups **Pairwise ***within the group

Regarding IL-4 level, there was a significant difference in the mean level between groups (*p* < 0.001) at 4 weeks post-treatment i.e., the diabetic treated group had a higher IL-4 mean level (265.5 ± 15.8 pg/ml) compared to both healthy control (88.7 ± 3.6 pg/ml, *p* < 0.001) and diabetic control (96.3 ± 8.2 pg/ml, *p* < 0.001). Contrarily, an insignificant difference was found between both control groups (*p* = 0.210). Further, at 8 weeks post-treatment, there was a significant difference (*p* < 0.001) in the mean IL-4 level between groups i.e., Diabetic treated group had higher IL-4 level (598.2 ± 7.8 pg/ml) compared to healthy control (92.8 ± 4.6 pg/ml, *p* < 0.001) and diabetic control (106.4 ± 5.9 pg/ml, *p* < 0.001). In contrast, an insignificant difference was between both controls (*p* = 0.159). For within-group comparisons, the increase in IL-4 was only significant for the diabetic-treated group (*p* < 0.001) (Fig. 2).

Respecting IL-10 levels, there was a significant difference in the mean level between groups (*p* < 0.001) at 4 weeks post-treatment i.e., the diabetic treated group had a higher IL-10 mean level (96.2 ± 34.8 pg/ml) compared to both healthy control (22.8 ± 6.4 pg/ml, *p* < 0.001) and diabetic control (36.3 ± 4.7 pg/ml, *p* < 0.001).

**Fig. 3:**
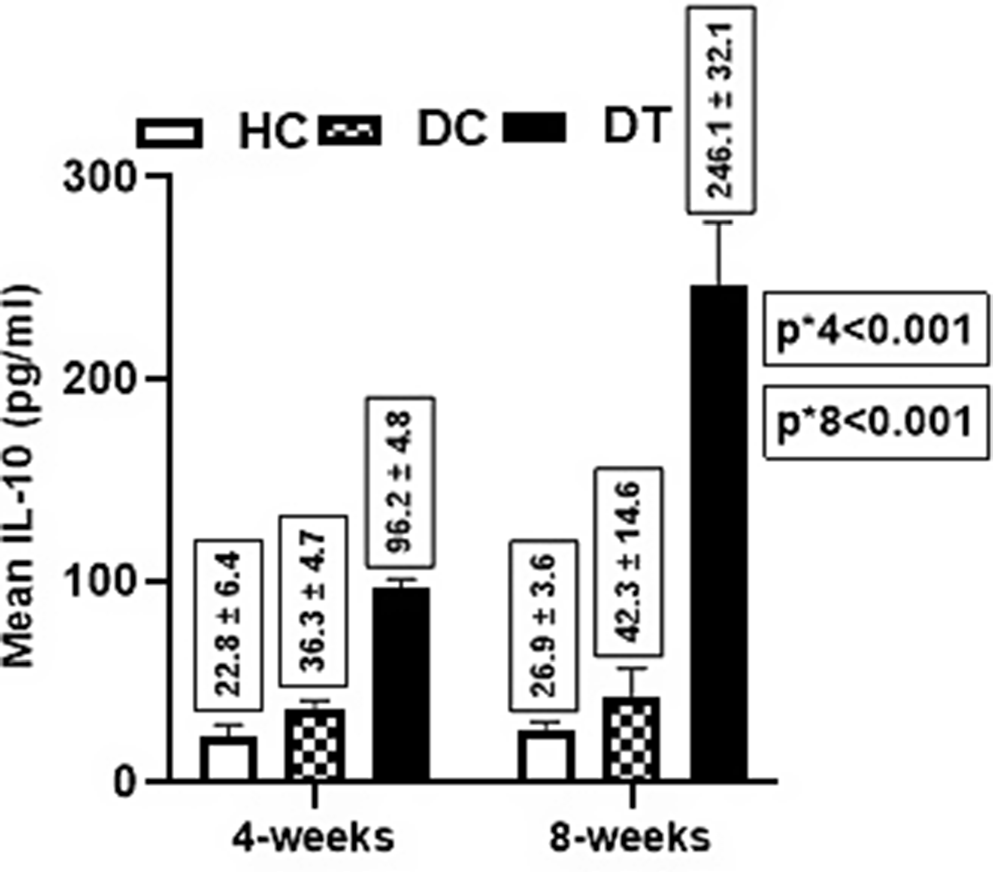
Effect of SEA on the IL-10 Level between Groups. P-value** 1 vs. 2 = 0.201 2 vs. 3 < 0.001 1 vs. 3 < 0.001 P-value*** = 0.751 = 0.464 < 0.001. A two-way ANOVA test was used to compare the mean difference *between groups **Pairwise ***within the group

Contrarily, an insignificant difference was found between both control groups (*p* = 0.586). Further, at 8 weeks post-treatment, there was a significant difference (*p* < 0.001) in the mean IL-10 level i.e., Diabetic treated group had higher IL-10 level (246.1 ± 32.1 pg/ml) compared to healthy control (26.9 ± 3.6 pg/ml, *p* < 0.001) and diabetic control (42.3 ± 14.6 pg/ml, *p* < 0.001). In contrast, an insignificant difference was between both controls (*p* = 0.201). For within-group comparisons, the increase in IL-10 was only significant for the diabetic-treated group (*p* < 0.001) (Fig. 3).

As shown in Table 3, there was a significant difference in the mean insulin level between groups (*p* < 0.001) at 4 weeks post-treatment i.e., the healthy control group had a higher mean level (18.7 ± 5.7 mIU/mL) compared to the diabetic control (7.2 ± 1.9 mIU/mL, *p* < 0.001) and diabetic treated (12.9 ± 3.1 mIU/mL, *p* = 0.002). Likewise, there was a significant difference between both diabetic groups (*p* < 0.001). Further, at 8 weeks post-treatment, the healthy control group had a higher mean level (22.3 ± 4.6 mIU/mL) compared to the diabetic control (6.5 ± 2.7 mIU/mL, *p* < 0.001) and diabetic treated (17.6 ± 6.1 mIU/mL, *p* = 0.034). Likewise, there was a significant difference between both diabetic groups (*p* < 0.001). For within-group comparisons, an increase in insulin level was only significant for the diabetic-treated group (*p* = 0.018).

**Table 3:** Effect of SEA treatment on the serum insulin level

	Healthy Control (1) (*n* = 10)	Diabetic Control (2) (*n* = 20)	Diabetic Treated (3) (*n* = 20)	*P*-value*
Insulin (mIU/mL)				
• 4-weeks Post-	18.7 ± 5.7	7.2 ± 1.9	12.9 ± 3.1	< 0.001
P-value**	1 vs. 2 < 0.001	2 vs. 3 < 0.001	1 vs. 3 = 0.002	
• 8-weeks Post-	22.3 ± 4.6	6.5 ± 2.7	17.6 ± 6.1	< 0.001
P-value**	1 vs. 2 < 0.001	2 vs. 3 < 0.001	1 vs. 3 = 0.034	
P-value***	= 0.168	= 0.357	= 0.018	

A two-way ANOVA test was used to compare the mean difference *between groups **Pairwise ***within the group

Regarding the blood glucose level, there was a significant difference in the mean blood glucose level between groups (*p* < 0.001) at 4 weeks post-treatment i.e., the healthy control group had a lower mean level (92.1 ± 3.7 mg/dl) compared to the diabetic control (225.3 ± 31.6 mg/dl, *p* < 0.001) and diabetic treated (184.2 ± 15.6 mg/dl, *p* < 0.001). Likewise, there was a significant difference between both diabetic groups (*p* < 0.001). Further, at 8 weeks post-treatment, the diabetic control group had a higher mean level (310.1 ± 28.7 mg/dl) compared to the healthy control (85.6 ± 6.4 mg/dl, *p* < 0.001) and the diabetic treated (125.4 ± 18.2 mg/dl, *p* < 0.001). Oppositely, an insignificant difference was found between healthy control and diabetic-treated groups (*p* = 0.279). For within-group comparisons, reduction in blood glucose level was significantly evident for the diabetic-treated group (*p* = 0.028). Notably, the increase in the blood glucose level was significantly evident for the diabetic control group (*p* = 0.004) (Table 4).

**Fig. 4:**
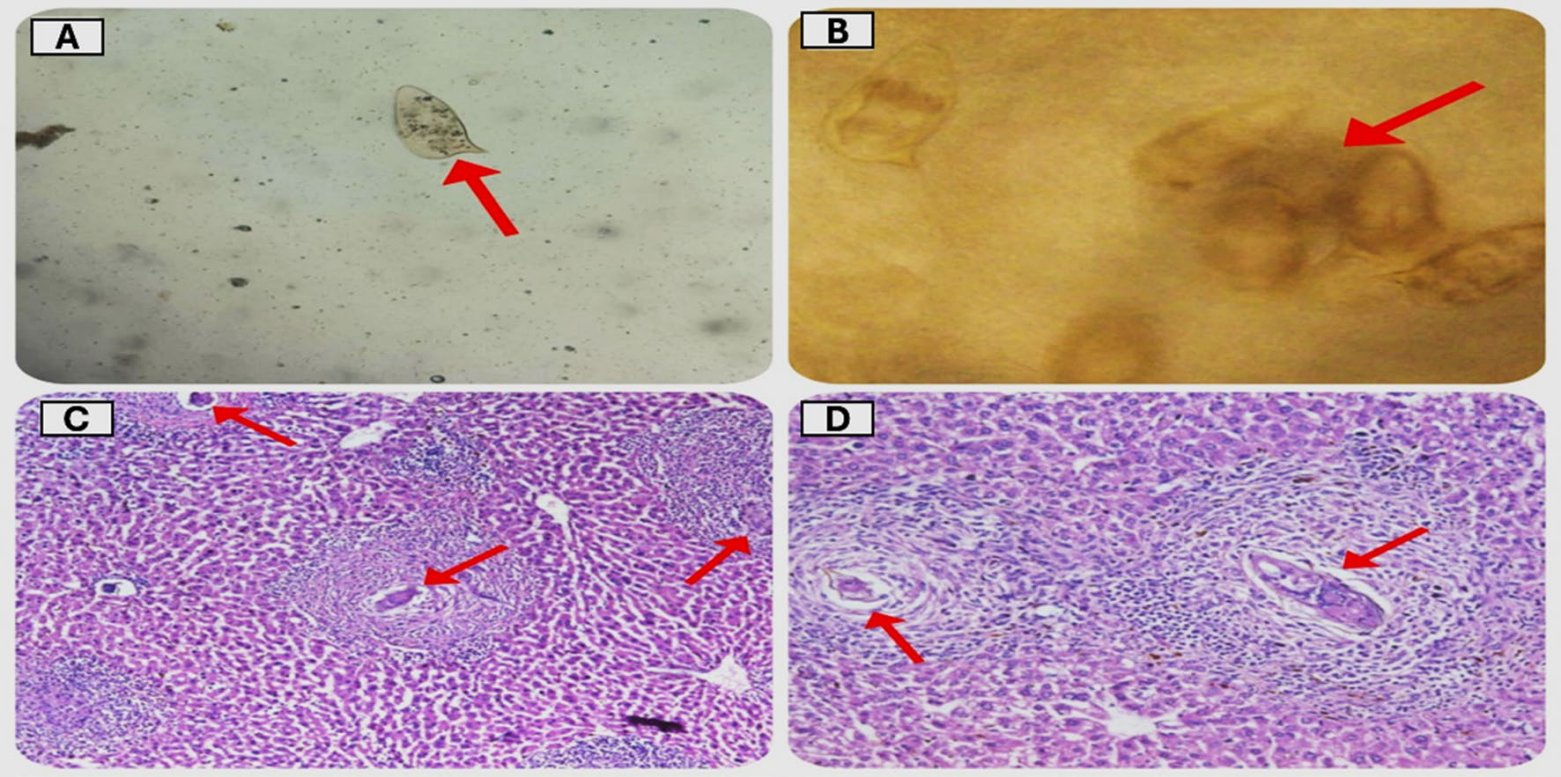
Microscopic examination of fecal pellets using (**A**) Direct wet mount and (**B**) Formol-ether concentration methods showed viable *Schistosoma mansoni* eggs (Red arrow). C&D. Histopathological Sections of rabbits’ liver stained with H&E showed multiple granulomas surrounding viable *Schistosoma mansoni* eggs (Red arrow) (magnification: X 40 & X 200)

Fig. 4 illustrates the presence of viable *Schistosoma mansoni* eggs, as determined through parasitological examination of fecal pellets collected from infected rabbits, and histopathological examination of liver sections from the same subjects

**Table 4:** Effect of SEA treatment on the serum blood glucose level

	Healthy Control (1) (*n* = 10)	Diabetic Control (2) (*n* = 20)		Diabetic Treated (3) (*n* = 20)	*P*-value*
Blood Glucose (mg/dL)					
• 4-weeks Post-	92.1 ± 3.7	225.3 ± 31.6		184.2 ± 15.6	< 0.001
P-value**	1 vs. 2 < 0.001	2 vs. 3 < 0.001		1 vs. 3 = 0.002	
• 8-weeks Post-	85.6 ± 6.4	310.1 ± 28.7		125.4 ± 18.2	< 0.001
P-value**	1 vs. 2 < 0.001	2 vs. 3 < 0.001		1 vs. 3 = 0.297	
P-value***	= 0.881	= 0.004		= 0.028	

A two-way ANOVA test was used to compare the mean difference *between groups **Pairwise ***within the group

## Discussion

The potential effectiveness of helminth infections and their derivative compounds in preventing T1D in a mouse model has been proven [15]. Multiple sclerosis (MS), Crohn’s disease, and T1D are among the Th1-mediated autoimmune disorders that have been demonstrated to be prevented by schistosomiasis infection and *Schistosoma*-derived antigens [16].

It is hypothesized that most autoimmune illnesses are caused by an imbalance in the Th1/Th2 immunological ratio, which results from an overpolarization of the Th1 immune response [17]. The polarization of the Th2 response and the counter-regulation of the Th1 response are intriguingly highly conserved aspects of helminth infection [6]. SEA is a complex mixture of phosphate-buffered saline-soluble compounds produced from mechanically ruptured eggs. It is extremely antigenic and can trigger a significant immune response [18].

Helminths such as *Taenia crassiceps*,* Strongyloides venezuelensis*,* filarial worms*,* Schistosoma spp.*,* Trichinella spiralis and Heligmosomoides polygyrus* have been reported to prevent diabetes in murine models [19].

To investigate the underlying mechanism, we selected several key markers: Tumor Necrosis Factor Alpha (TNF-α), a pro-inflammatory cytokine and marker of the Th1 immune response. IL-4 and IL-10, which serve as markers of the Th2 response. Forkhead Box P3 (FOXp3), a specific marker for T regulatory cells (Tregs). Transforming Growth Factor Beta (TGF-β), which is critical for the development and maintenance of Tregs, maintaining immune tolerance and preventing autoimmune diseases [20].

Our research showed that the diabetic-treated group had a higher FOXp3 and TGF-β mean level compared to control groups (*p* < 0.001).

These findings align with previous reports that *Schistosoma mansoni* SEA prevents diabetes in nonobese diabetic (NOD) mice by causing functional changes in antigen-presenting cells and increasing Th2 and Treg responses. They also showed that SEA treatment increases bioactive TGF-β on T cells, which leads to an expansion of FOXp3 + Tregs, which may play a role in SEA-mediated diabetes prevention in conjunction with Th2 cells [21].

Another study has reported that mice infected with *S. mansoni* showed elevated levels of IL-5, IL-10, and FOXp3 when T1D was induced, which demonstrated evidence of a shift in the immune response toward Th2 and Treg pathways [12]. Treatment with SEA increases the bioactivity of transforming growth factor-β, which is crucial for the effective Th2 response to SEA as well as for Treg proliferation. Although antigen-specific Treg transfer may delay and even cure type 1 diabetes [22], Treg deficiency or diminished suppressive function may be a factor in the development of diabetes in NOD mice [23].

In Type 1 diabetes mellitus (T1D) a significant increase in the expression of interleukin-10 (IL-10) and tumor necrosis factor-alpha (TNF-α) genes has been observed. Since the current T1D treatment relies primarily on exogenous insulin, there is a need for alternative therapeutic strategies. Inflammatory biomarkers hold promise for offering novel insights and opportunities for advancing the therapeutic approach to patient care [24].

The understanding of TNF-α signalling mechanisms has advanced significantly and has been effectively applied to the treatment of immune-mediated diseases. Clinically approved TNF-α inhibitors have demonstrated remarkable efficacy in managing various autoimmune diseases [25]. Our investigation showed a significantly decreased level of TNF-α in the diabetic-treated group (*p* < 0.001).

At 4 and 8 weeks, respectively, the Th2 markers IL 4 and IL 10 demonstrated a large and highly significant increase. Parasitic helminths control the host’s immune system and trigger Th2 and Tregs immunological responses, which may delay the onset of diabetes [26]. The adoptive transfer of dendritic cells (DC) genetically modified to express interleukin-4 (IL-4) significantly reduces the onset of diabetes in both normoglycemic and prediabetic nonobese diabetic (NOD) mice. It produces shifting from Th1 to Th2, delays the progression to hyperglycemia, decreases islet inflammation and enhances regulatory T-cell activity [27].

Helminth infection with Heligmosomoides polygyrus prevents type 1 diabetes (T1D) in nonobese diabetic mice by enhancing IL-10 production from CD4(+) T cells, with protection lasting up to 40 weeks [28].

In streptozotocin-induced T1D in wild-type mice, *Schistosoma mansoni* infection inhibits the breakdown of pancreatic islets and hyperglycemia through increased Arg-1 and Ym1 expression rather than through processes that rely on Treg, IL-4, IL-13, and IL-10 [29].

There is some evidence that helminth worms control interleukin-10, an anti-inflammatory cytokine that is controlled by T helper cells, and this lowers the risk of autoimmune illness by assisting in the maintenance of low levels of intestinal inflammation. Accordingly, disease prevention and treatment may benefit from similar IL-10 pathway modification through the administration of parasitic extract or medication development [30].

Similar studies documented the Th2 response of SEA in experimental mice in the form of elevated serum levels of IL-10 [20, 31]. IL-10 has also been shown to significantly reduce the incidence of diabetes and delay the onset of disease when administered daily and subcutaneously to NOD mice. The IL-10 released by Th2 prevents Th1 from producing pro-inflammatory cytokines. This balances the system to a non-allergic, non-autoimmune state and counteracts the human body’s excessive immune response.

Tregs can modify immune responses by suppressing effector T cell responses or secreting the cytokines IL-10 and TGF-β to create immunological tolerance, even though IL-10 may be the most promising of the anti-inflammatory interleukins being researched to prevent type 1 diabetes [32]. The incidence of T1DM in rodents is reduced by *Schistosoma mansoni* infection or its adult/egg antigens, and numerous studies have examined similar outcomes in other T1DM animal models with *Schistosoma japonicum* infection [33].

Our research showed that the diabetic control group had significantly higher blood glucose levels than the healthy control group (*P* < 0.001). Glucose levels in the treated diabetic group were significantly lower than those in the non-treated diabetic control (*P* < 0.001). This implies that in mice with SPZ-induced diabetes, *S. mansoni* SEA can prevent T1D. This was consistent with earlier research that showed that SEA treatment greatly reduced the percentage incidence of diabetes [9, 34, 35].

The diabetic control group’s insulin levels were considerably lower than those of the healthy group (*p* < 0.001). When compared to the diabetic control group, the diabetic-treated group experienced a significant drop (*P* = 0.03). Our results were consistent with other research that reported a potential immune effect of SEA in treating and preventing the development of T1D, evidenced by lower blood glucose and higher blood insulin levels [12, 36].

The *Schistosoma mansoni* infection plays a protective role in the prevention of autoimmune-mediated insulin-dependent diabetes mellitus (IDDM) in mice induced by multiple low doses of streptozotocin (STZ). The infection significantly mitigated hyperglycemia, preserved pancreatic islet structure, and maintained a higher number of β-cells compared to non-infected diabetic mice. The protective mechanism appears to be mediated by a shift from a Th1-dominant immune response (IFN-γ) to a Th2/Treg-associated profile (IL-4, IL-5, IL-10, and TGF-β), leading to the suppression of STZ-induced β-cell destruction [37].

Our findings generally imply that *S. mansoni* SEA can be used to treat T1D by enhancing Th2-type and Tregs immune responses. At 4 weeks post-treatment, immunological markers showed significant modulation, reflecting potential therapeutic effects. A moderate increase in FOXp3 levels suggests stimulation of regulatory T cells, which might contribute to the reduction of inflammation. Concurrently, the observed increase in TGF-β highlights an upregulation of immunosuppressive pathways, which could counteract inflammation effectively. A reduction in TNF-α, a key pro-inflammatory cytokine, represents a positive outcome, as it implies dampened inflammatory activity. Additionally, the increases in IL-4 and IL-10 levels point to a shift towards an anti-inflammatory state, promoting a favourable immunological environment that could benefit diabetes management by mitigating chronic inflammation.

At 8 weeks post-treatment, immunological and metabolic markers reveal sustained effects, some of which warrant cautious interpretation. Immunological indicators exhibited notable alteration four weeks after treatment, suggesting possible therapeutic benefits. In the absence of other anti-inflammatory cues, an increase in FOXp3 levels may indicate a proliferation in regulatory T cells, which could lead to decreased inflammation. At the same time, the observed increase in TGF-β indicates an activation of immunosuppressive pathways, which may successfully combat inflammation. Since it suggests lessened inflammatory activity, a decrease in TNF-α, a crucial pro-inflammatory cytokine, is a good result. Furthermore, a shift towards an anti-inflammatory state is indicated by the increases in IL-4 and IL-10 levels, which could help manage diabetes by reducing chronic inflammation and creating a favourable immunological environment.

## Conclusions

Our findings showed significant increases in TGF-ß, IL-4, IL-10, and FOXp3 Tregs in SPZ-induced diabetic mice after SEA therapy. So SEA can prevent type 1 diabetes by improving regulatory T cells and the T helper 2 cell immune response. Future research should focus on the long-term efficacy of SEA, its molecular mechanisms of action, potential for beta-cell protection and regeneration, suitability in combination therapies, translational studies in humans, and broader applications in other autoimmune diseases.

## Data Availability

No datasets were generated or analysed during the current study.
